# Supplementary material for: PAM recognition by miniature CRISPR–Cas12f nucleases triggers programmable double-stranded DNA target cleavage
Source: Nucleic Acids Res. 2020 Apr 4;48(9):5016–23. doi: 10.1093/nar/gkaa208 (PMC7229846; doi:10.1093/nar/gkaa208)
Supplement: gkaa208_Supplemental_Files [file gkaa208_supplemental_files.zip › 2020_SM_revised.pdf]

Supplementary Information for

PAM recognition by miniature CRISPR-Cas12f nucleases triggers programmable double-stranded DNA target cleavage

Tautvydas Karvelis, Greta Bigelyte, Joshua K. Young, Zhenglin Hou, Rimante Zedaveinyte, Karolina Budre, Sushmitha Paulraj, Vesna Djukanovic, Stephen Gasior, Arunas Silanskas, Česlovas Venclovas, Virginijus Siksnys

This PDF file includes:

Supplementary Tables S1-S2

Supplementary Figures S1-S15

Legend Supplementary Data S1

SI References

Other supplementary materials for this manuscript include the following:

Supplementary Data S1

Supplementary Table S1. Cas12f proteins used in this study.

**Name**	**Previous name**	**Size (aa)**	**Molecular mass (kDa)**	**Protein id (NCBI)**	**Organism**	**Scaffold accession (NCBI)**
Mi1Cas12f2	Cas14b4*	544	63.9	OIO21000.1	Candidatus Micrarchaeota archaeon	MK005740.1
Un1Cas12f1	Cas14a1*	529	61.5	QBM01166.1	uncultured archaeon	MK005734
Un2Cas12f1	Cas14a3*	500	58.4	QBM01093.1	uncultured archaeon	MK005732
Mi2Cas12f2	Cas14b17**	586	69.4	RLG21245.1	Candidatus Micrarchaeota archaeon	QMVG01000004.1
AuCas12f2	Cas14b18**	603	69.9	RJP56748.1	Candidatus Aureabacteria bacterium	QZJZ01000091.1
PtCas12f1[+]	C2c10[++]	424	49.5	WP_064552366.1	*Parageobacillus thermoglucosidasius*	NZ_LXMA01000038.1
AsCas12f1[+]	C2c10[++]	422	48.7	WP_109431741.1	*Acidibacillus sulfuroxidans*	NZ_MPDK01000047.1
RuCas12f1[+]	C2c10[++]	440	51.2	WP_117896622.1	Unclassified *Ruminococcus*	NZ_QTWX01000005.1
SpCas12f1[+]	C2c10[++]	497	56.9	WP_054696859.1	*Syntrophomonas palmitatica*	NZ_BBCE01000017.1
CnCas12f1[+]	C2c10[++]	497	58.5	WP_120361969.1	*Clostridium novyi*	NZ_CP029458.1

* Identified by Harrington *et al*. 2018 (1).

** Identified by BLAST alignments against the NCBI NR database. Numbering is continued from that described in Harrington *et al*. 2018 (1).

[+] Type V-U3 nuclease identified by PSI-BLAST.

[++] Originally denoted name by Shmakov *et al*. 2017 (2).

Supplementary Table S2. Plasmids used in this study.

Plasmid name	Description	Link
PV424	Mi1Cas12f2 engineered and intact locus (native expression)	https://benchling.com/s/seq-uDaLUdexDYQQSz7QDMXF
PV477	Disrupted Mi1Cas12f2 (native expression)	https://benchling.com/s/seq-y7Fh5ryHUNNX7R3sDYMa
R-652	Mi1Cas12f2 intact locus (T7 expression)	https://benchling.com/s/seq-DNGdXS8DCheZt5Nv6kMu
R-656	Mi1Cas12f2 minimal locus (T7 expression)	https://benchling.com/s/seq-kUL4zwpBZDDJIwIwwLBT
R-658	Minus Mi1Cas12f2 (T7 expression)	https://benchling.com/s/seq-A2E4WYBe0vLNbHiWQ2py
pLBH531	10×His-MBP-Un1Cas12f1 (Cas14a1) expression	https://www.addgene.org/112500/
pLBH545	Un1Cas12f1 (Cas14a1) locus (tetracycline inducible expression)	https://www.addgene.org/112501/
pGB53	Un1Cas12f1 and sgRNA expression (pLBH545-based; T7 and tetracycline inducible expression)	https://benchling.com/s/seq-QoHpAbpI97JLSnhzh8Mn
pGB49	10×His-MBP- Un1Cas12f1 D326A expression (pLBH531-based)	https://benchling.com/s/seq-qpUDPxM6IBhTJFXpNVUJ
pGB50	10×His-MBP- Un1Cas12f1 D510A expression (pLBH531-based)	https://benchling.com/s/seq-W1miOvRPGZ44fgZnV0Nn
pUn2Cas12f1-pETduet-1	Un2Cas12f1 intact locus (T7 expression)	https://benchling.com/s/seq-6PdzgqR721Th21GAzyM7
pMi2Cas12f2-pETduet-1	Mi2Cas12f2 intact locus (T7 expression)	https://benchling.com/s/seq-QJzxQ4p6OonoQ8ZszpF7
pAuCas12f2-pETduet-1	AuCas12f2 intact locus (T7 expression)	https://benchling.com/s/seq-6lJN8dk7ZTmbimkqw7Dg
pPtCas12f1-pETduet-1	PtCas12f1 intact locus (T7 expression)	https://benchling.com/s/seq-qaU17VDPHblLUii1K5QZ
pAsCas12f1-pETduet-1	AsCas12f1 intact locus (T7 expression)	https://benchling.com/s/seq-JWWCYPn66yxJl12WMBMX
pRuCas12f1-pETduet-1	RuCas12f1 intact locus (T7 expression)	https://benchling.com/s/seq-XFA0y65xFCT57R719yVV
pSpCas12f1-pETduet-1	SpCas12f1 intact locus (T7 expression)	https://benchling.com/s/seq-7LSUEFWIvEQk2AMpvsGA
pCnCas12f1-pETduet-1	CnCas12f1 intact locus (T7 expression)	https://benchling.com/s/seq-92ITTuoZVYrJfN1hYgX1
pTZ57	7N PAM plasmid library	https://benchling.com/s/seq-nu2IvfXbn7smVQ7T6MYi
pGB33	Mi1Cas12f2 target plasmid (pUC18-based)	https://benchling.com/s/seq-iYcV6jflHOPbUxMdCGs9
pGB40	Un1Cas12f1 target plasmid (pUC18-based)	https://benchling.com/s/seq-XGplLg5diY1G7BwBDHXn
pGB41	Un1Cas12f1 ΔPAM (target plasmid) (pUC18-based)	https://benchling.com/s/seq-QS7mA2qo4e3JRlBvKsXb

pGB42	Un1Cas12f1 non-target plasmid (pUC18-based)	https://benchling.com/s/seq-rXjk6jlGlmPUSbb2GeT9
pTHSSe_1	Un1Cas12f1 and Mi1Cas12f2 non-target plasmid (pSC101 ori)	https://www.addgene.org/109233/
pGB43	Un1Cas12f1 target plasmid (pTHSSe_1-based)	https://benchling.com/s/seq-Xt674hLUcgdIn2BO0tJ1
pKP17	Mi1Cas12f2 target plasmid (pTHSSe_1-based)	https://benchling.com/s/seq-g4SqeTyluv6C5FEwfmfS
pSG4K5	Un2Cas12f1, Mi2Cas12f2, AuCas12f2, PtCas12f1, AsCas12f1, RuCas12f1, SpCas12f1 and CnCas12f1 non-target plasmid (pSC101 ori)	https://www.addgene.org/74492/
pKP8	Un2Cas12f1, Mi2Cas12f2, AuCas12f2, PtCas12f1 and AsCas12f1 target plasmid (pSG4K5-based)	https://benchling.com/s/seq-P3Bzoe526M7vh0Cv3mtD
pKP9	RuCas12f1 target plasmid (pSG4K5-based)	https://benchling.com/s/seq-8JkUE7ndI46oxuapcPWS
pKP10	SpCas12f1 target plasmid (pSG4K5-based)	https://benchling.com/s/seq-dIsHC0DVhRF4z8bLWdbw
pKP11	CnCas12f1 target plasmid (pSG4K5-based)	https://benchling.com/s/seq-6t7yhm7Vs6FwSj2N2qOu

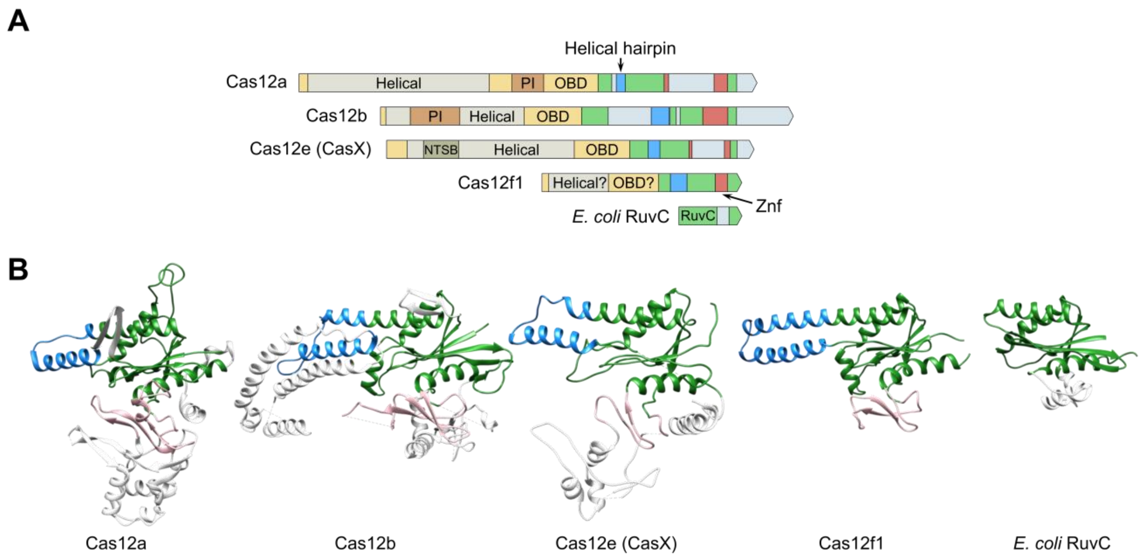

Supplementary Figure S1. Protein architecture and C-terminal RuvC domain structural comparisons between Cas12 dsDNA effectors and Cas12f. Comparison includes Cas12a (PDB id: 5xut), Cas12b (PDB id: 5wti), Cas12e (CasX) (PDB id: 6ny2), Un1Cas12f1 (Cas14a1) (model), and *E. coli* RuvC (PDB id: 1hjr). Un1Cas12f1 region homologous to RuvC was identified using HHpred (3). Structural model for Un1Cas12f1 sequence region was generated by the Rosetta comparative modeling protocol (4) using the structure of Cas12b (PDB id: 5WTI) as a modeling template. Five models generated by Rosetta were evaluated using the VoroMQA web server (5), designed to assess three-dimensional structures of proteins and protein complexes. The best-scoring model was selected and subjected to the protein structure refinement using the GalaxyRefine2 web server with standard refinement parameters (6). Multiple refined structural models returned by GalaxyRefine2 were again scored with VoroMQA and the best-scoring one was selected as the final model. (**A**) Organization of protein domains and relative size comparison between known type V dsDNA effectors and Cas12f1. (**B**) C-terminal RuvC domain of known type V dsDNA effectors, Cas12f1, and *E. coli* RuvC. The common RuvC core is colored green in all the structures. Helical hairpin common to Cas proteins, but absent from the *E. coli* RuvC is shown in blue. Common zinc finger motif (CasX and Cas12f1) or a zinc finger-like motif (Cas12a and Cas12b) is shown in pink. PAM-interacting (PI) domain is shown in light brown, nucleotide binding domain (OBD) in yellow and non-target strand binding (NTSB) residues in moss-green. Other structure-specific motifs are shown in grey.

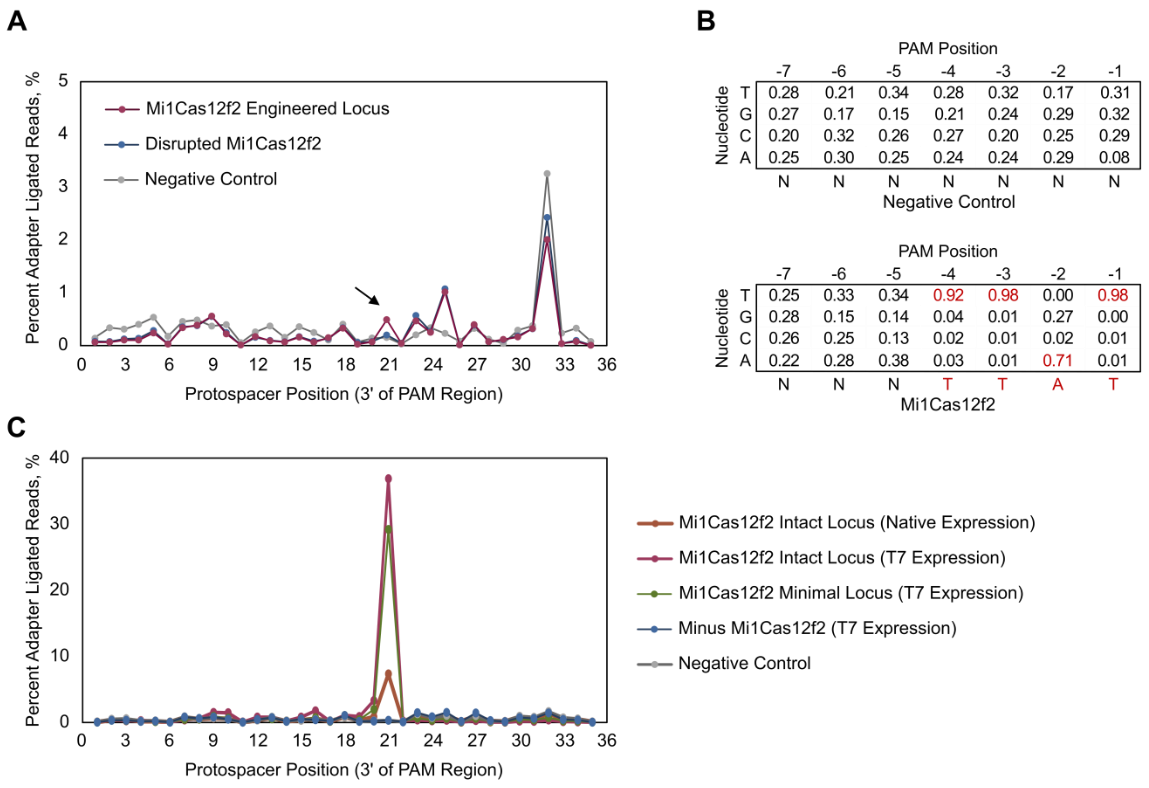

Supplementary Figure S2. Mi1Cas12f2 dsDNA recognition and cleavage. (**A**) Relative to the negative controls, the Mi1Cas12f2 locus engineered to target a PAM library (36 nt spacers) produced a spike in the recovery of protospacer fragments ligated to an adapter just after the 21st position 3' of the PAM region. (**B**) PAM sequences that supported cleavage generated a position frequency matrix (PFM) exhibiting preferences for T and A bps 5' of the gRNA target. As a reference, a PFM at the same position in the lysate only control was also calculated. (**C**) dsDNA plasmids containing a PAM and gRNA target showed an even greater enrichment in the recovery of adapters ligated just after the 21st position, especially for reactions where expression was enhanced with a T7 promoter. Experiments deleting *cas1*, *cas2*, and *cas4* genes (Mi1Cas12f2 Minimal Locus) and the *cas12f2* gene itself (Minus Mi1Cas12f2) confirmed that Mi1Cas12f2 was the only protein required for the observed dsDNA target recognition and cleavage.

A

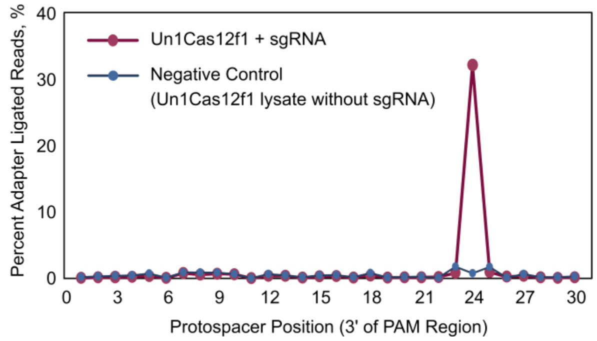

B

PAM Position

Nucleotide	-7	-6	-5	-4	-3	-2	-1
T	0.20	0.33	0.33	0.93	0.97	0.88	0.08
G	0.30	0.14	0.19	0.01	0.00	0.04	0.46
C	0.28	0.22	0.20	0.03	0.03	0.06	0.01
A	0.22	0.32	0.29	0.03	0.00	0.03	0.45
	N	N	N	T	T	T	R

Un1Cas12f1

Supplementary Figure S3. Un1Cas12f1 (Cas14a1) dsDNA recognition and cleavage. (**A**) *E. coli* lysate containing Un1Cas12f1 and sgRNA targeting the PAM library (20 nt spacer) produced an enrichment in the recovery of protospacer adapter ligated fragments just after the 24th position 3’ of the PAM region relative to the negative control. (**B**) Displayed as a PFM, PAM sequences that supported cleavage showed a 5’ T-rich PAM.

A

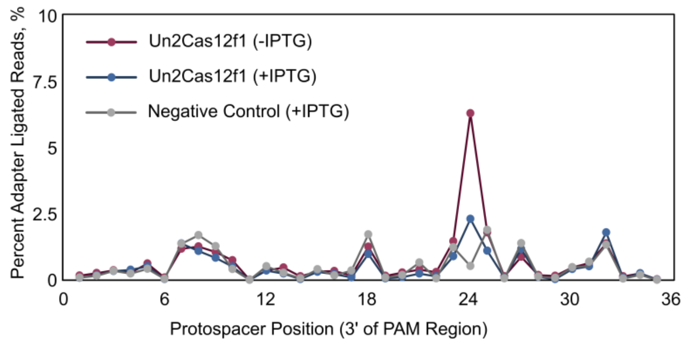

B

PAM Position

Nucleotide	-7	-6	-5	-4	-3	-2	-1
T	0.21	0.24	0.34	0.88	1.00	0.98	0.00
G	0.28	0.22	0.15	0.02	0.00	0.00	0.42
C	0.26	0.24	0.20	0.07	0.00	0.01	0.02
A	0.26	0.30	0.31	0.03	0.00	0.01	0.56
Un2Cas12f1	N	N	N	T	T	T	R

Supplementary Figure S4. Un2Cas12f1 dsDNA recognition and cleavage. (**A**) *E. coli* lysate from cells expressing the minimal CRISPR-Un2Cas12f1 locus modified to target the 7N PAM library (37 nt spacers) produced a spike in the recovery of adapter ligated fragments just after the 24th position 3’ of the PAM with and without induction of expression with IPTG. (**B**) Similar to Un1Cas12f1, a 5’ T-rich PAM was shown to coincide with the cleavage signal.

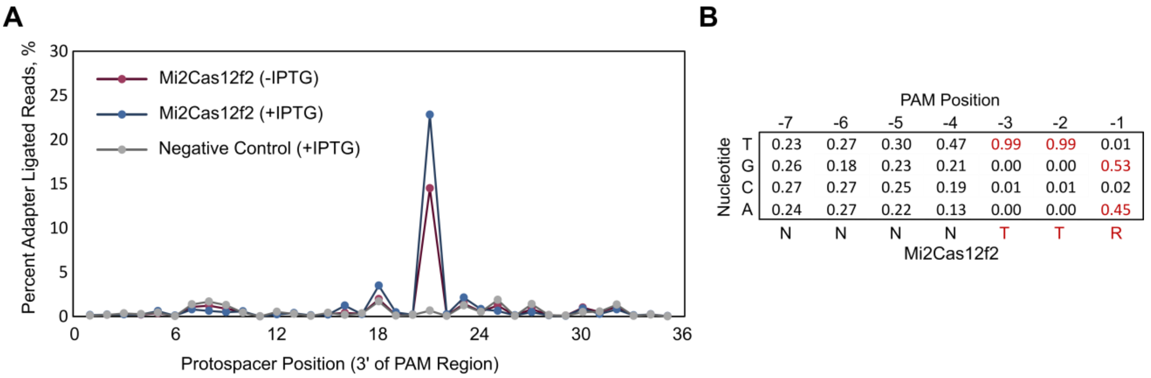

Supplementary Figure S5. Mi2Cas12f2 dsDNA recognition and cleavage. (**A**) *E. coli* lysate containing Mi2Cas12f2 protein and guide RNAs targeting the PAM library (39 nt spacers) produced an enrichment in the recovery of protospacer adapter ligated fragments just after the 21st position 3' of the PAM region relative to the negative control (with and without IPTG induction of expression). (**B**) Displayed as a PFM, fragments with an adapter ligated after the 21st position exhibited a strong bias towards 5'-TTR(A/G)-3' sequences in the PAM library.

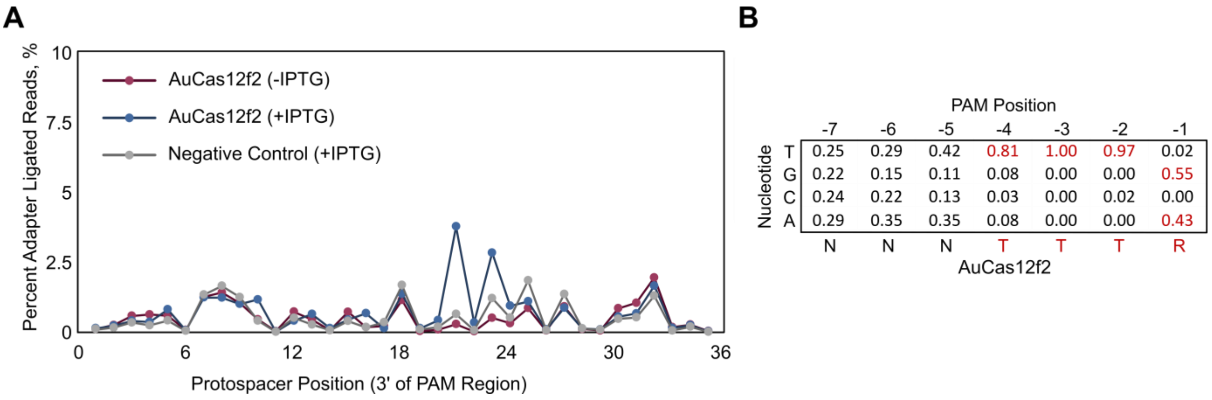

Supplementary Figure S6. AuCas12f2 dsDNA recognition and cleavage. (**A**) *E. coli* lysate containing AuCas12f2 and guide RNAs targeting the PAM library (38 nt spacers) produced an enrichment in the recovery of protospacer adapter ligated fragments just after the 21st and 23rd positions 3’ of the PAM region relative to the negative control. (**B**) Displayed as a PFM, fragments with an adapter ligated after the 21st position exhibited a strong bias towards 5’-TTTR(A/G)-3’ sequences in the PAM library.

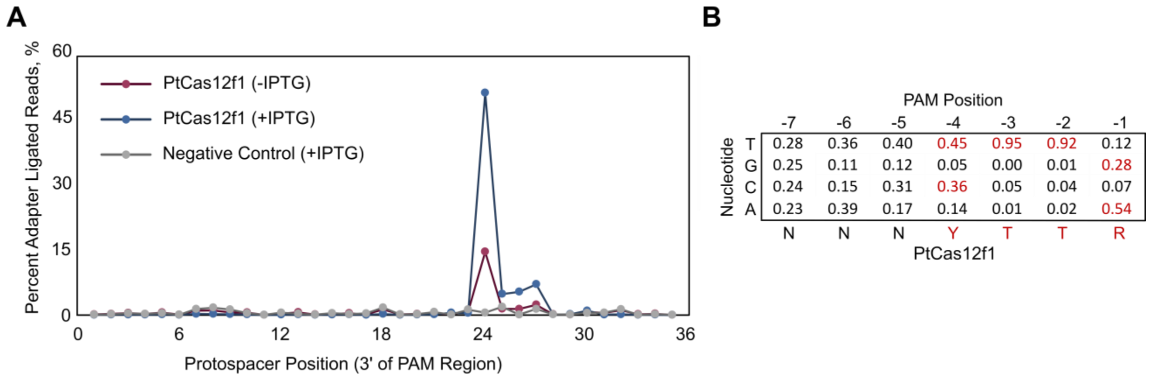

Supplementary Figure S7. PtCas12f1 dsDNA recognition and cleavage. (**A**) *E. coli* lysate from cells expressing the minimal CRISPR-PtCas12f1 locus modified to target the 7N PAM library (36 nt spacers) produced a spike in the recovery of adapter ligated fragments just after the 24th position 3' of the PAM with and without induction of expression with IPTG. (**B**) Displayed as a PFM, fragments with an adapter ligated after the 24th position showed preference for 5'-Y(T/C)TTR(A/G)-3' sequences in the PAM library.

A

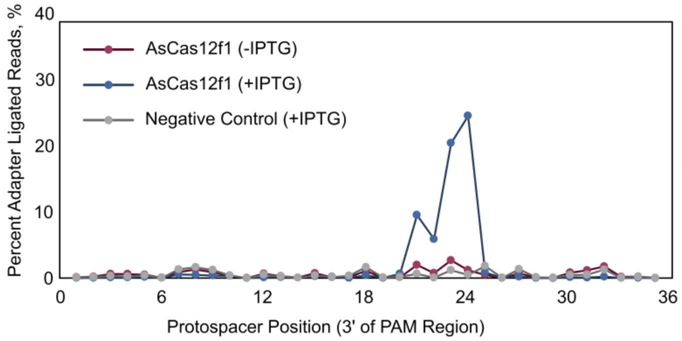

B

PAM Position

Nucleotide	-7	-6	-5	-4	-3	-2	-1
T	0.26	0.31	0.34	0.44	0.93	0.86	0.28
G	0.27	0.13	0.17	0.08	0.01	0.04	0.27
C	0.32	0.23	0.27	0.31	0.06	0.08	0.14
A	0.15	0.33	0.21	0.17	0.00	0.03	0.32
AsCas12f1	N	N	N	Y	T	T	N

Supplementary Figure S8. AsCas12f1 dsDNA recognition and cleavage. (**A**) Relative to the negative control, the AsCas12f1 locus engineered to target a PAM library (38 nt spacers) produced a spike in the recovery of protospacer fragments ligated to an adapter after the 21st position 3' of the PAM region. (**B**) Displayed as a PFM, fragments with an adapter ligated after the 21st position revealed a strong bias towards 5'-Y(T/C)TTN-3' sequences in the PAM library.

A

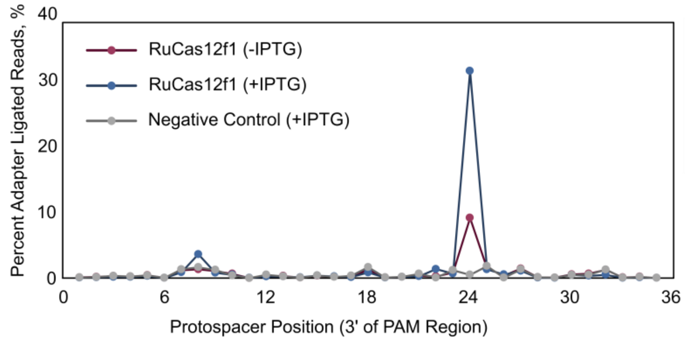

B

PAM Position

Nucleotide	-7	-6	-5	-4	-3	-2	-1
T	0.26	0.27	0.28	0.26	0.05	0.97	0.87
G	0.28	0.19	0.22	0.23	0.00	0.02	0.00
C	0.28	0.25	0.21	0.28	0.94	0.01	0.13
A	0.19	0.29	0.28	0.23	0.01	0.00	0.00
RuCas12f1	N	N	N	N	C	T	T

Supplementary Figure S9. RuCas12f1 dsDNA recognition and cleavage. (**A**) *E. coli* lysate containing RuCas12f1 protein and guide RNAs targeting the PAM library (33 nt spacers) produced an enrichment in the recovery of protospacer adapter ligated fragments just after the 24th position 3’ of the PAM region relative to the negative control (with and without IPTG induction of expression). (**B**) Displayed as a PFM, fragments with an adapter ligated after the 24th position demonstrated preference for 5’-CTT-3’ sequences in the PAM library.

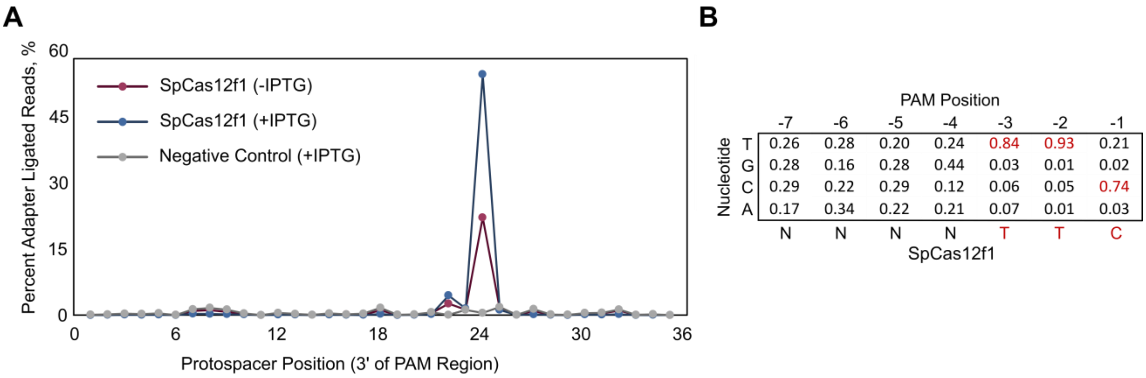

Supplementary Figure S10. SpCas12f1 dsDNA recognition and cleavage. (**A**) *E. coli* lysate from cells expressing the minimal CRISPR-SpCas12f1 locus modified to target the 7N PAM library (34 nt spacers) produced a spike in the recovery of adapter ligated fragments just after the 24th position 3' of the PAM with and without induction of expression with IPTG. (**B**) Displayed as a PFM, fragments with an adapter ligated after the 24th position exhibited a strong bias towards 5'-TTC-3' sequences in the PAM library.

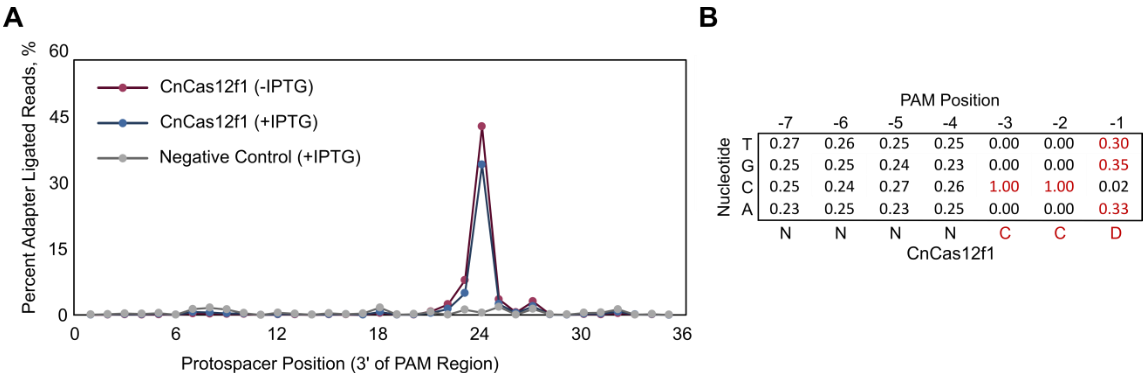

Supplementary Figure S11. CnCas12f1 dsDNA recognition and cleavage. (**A**) Relative to the negative controls, the CnCas12f1 locus engineered to target a PAM library (37 nt spacers) produced a spike in the recovery of protospacer fragments ligated to an adapter after the 24th position 3' of the PAM region. (**B**) Displayed as a PFM, fragments with an adapter ligated after the 24th position exhibited a strong bias towards 5'-CCD(T/G/A)-3' sequences in the PAM library.

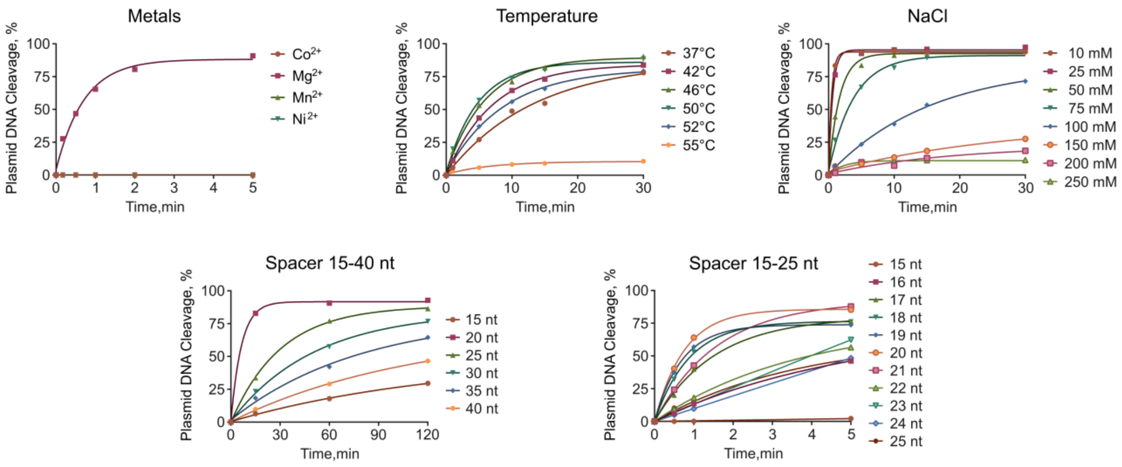

Supplementary Figure S12. Optimization of reaction conditions for Un1Cas12f1 (Cas14a1) RNP mediated plasmid DNA cleavage. Un1Cas12f1 RNP plasmid DNA cleavage was assayed by independently varying divalent metal ions, temperature, NaCl concentration and sgRNA spacer length. Other than in experiments testing sgRNA spacer length, Un1Cas12f1 RNP complexes were assembled using a sgRNA with a 20 nt spacer.

A

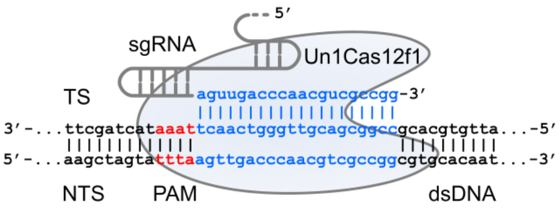

B

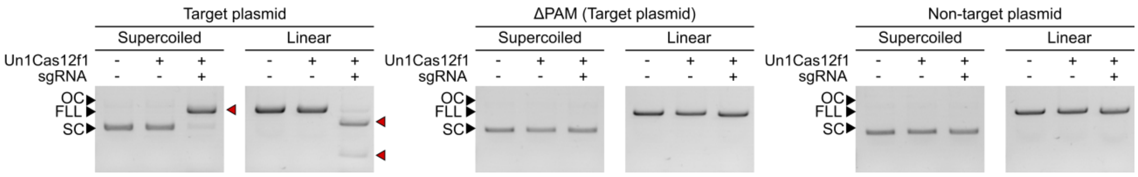

Supplementary Figure S13. Un1Cas12f1 (Cas14a1) RNP complex is a PAM-dependent dsDNA endonuclease. (**A**) Schematic representation of Un1Cas12f1 RNP complex with target DNA. (**B**) Un1Cas12f1 RNP complexes cleaved plasmid DNA targets in a PAM-dependent manner (left panel) requiring both PAM (center panel) and sgRNA recognition (right panel). Un1Cas12f1 RNP complexes were assembled using a sgRNA with a 20 nt length spacer. SC, OC, and FLL stand for supercoiled, open-circle, and full-length linearization, respectively. NTS and TS represent non-target strand and target strand, respectively.

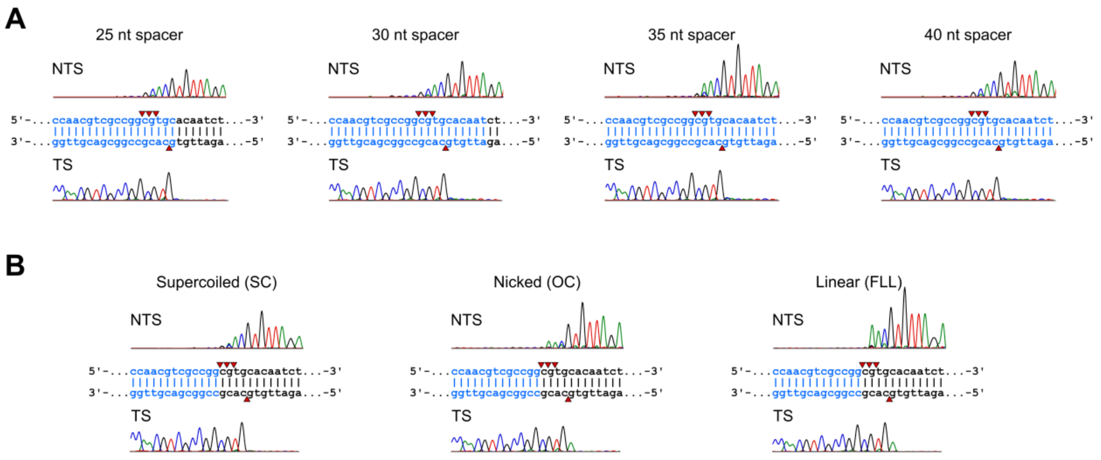

Supplementary Figure S14. Run-off sequencing of Un1Cas12f1 (Cas14a1) cleaved plasmid DNA. Plasmid DNA cleavage resulted in a double-stranded break centered around positions 20-24 bp 3' of the PAM. The cleavage pattern was independent of spacer length (**A**) and plasmid topology (**B**). NTS and TS represent non-target strand and target strand, respectively.

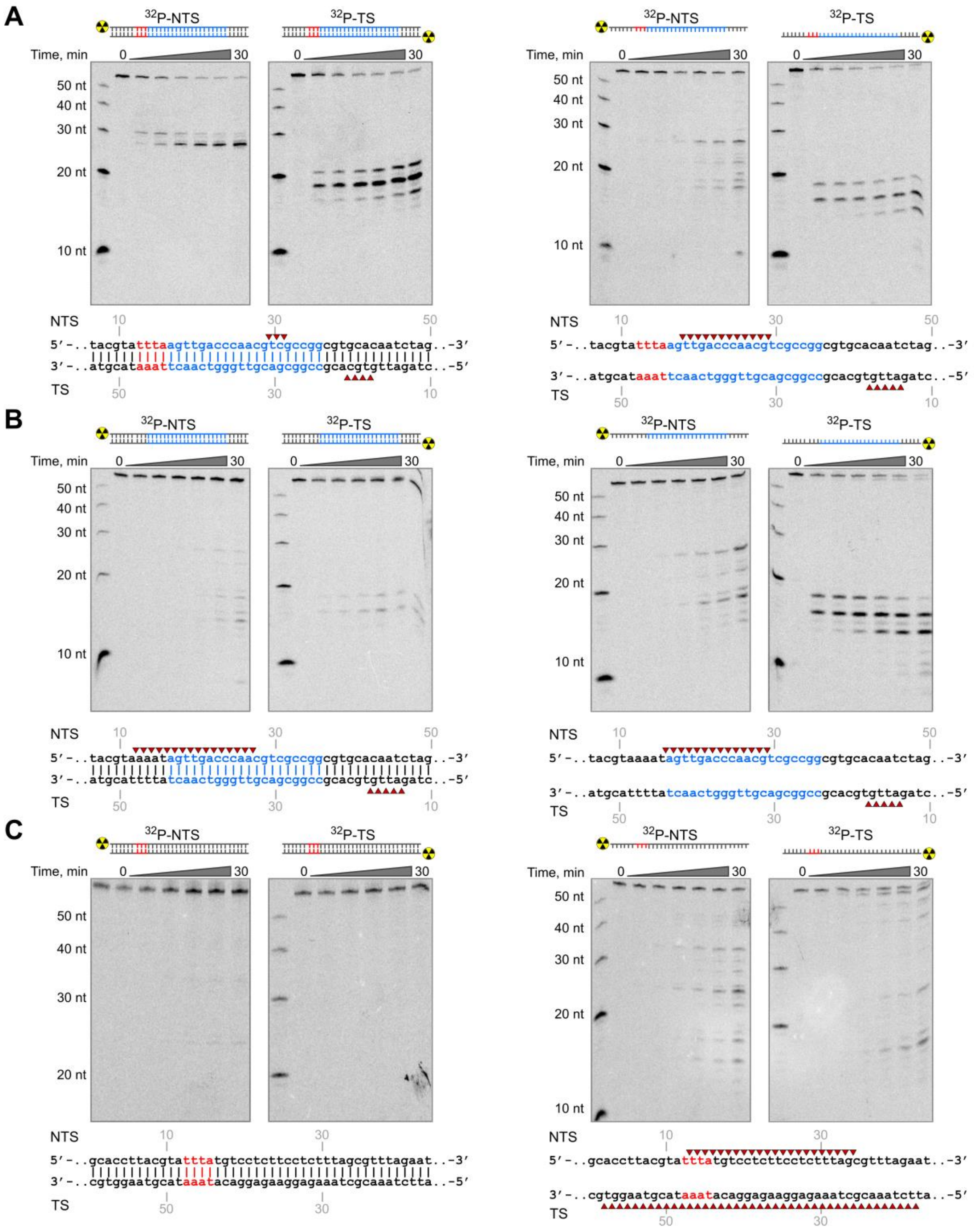

Supplementary Figure S15. Oligoduplex cleavage by Un1Cas12f1 (Cas14a1) RNP complex. (**A**) Purified Un1Cas12f1 RNP complexes cleaved radiolabeled dsDNA oligoduplexes containing a sgRNA target in a PAM-dependent manner generating a staggered cleavage pattern. dsDNA substrates without PAM (**B**) or target sequence (**C**) were not cleaved by the Un1Cas12f1 RNP complex. ssDNA substrates complementary to the sgRNA spacer sequence were also cleaved albeit in the PAM-independent manner (A and B). Un1Cas12f1 RNP complexes were assembled using a sgRNA with a 20 nt length spacer. NTS and TS represent non-target strand and target strand, respectively.

Supplementary Data S1 (separate file). DNA, RNA and protein sequences used in this study.

References

1. Harrington,L.B., Burstein,D., Chen,J.S., Paez-Espino,D., Ma,E., Witte,I.P., Cofsky,J.C., Kyrpides,N.C., Banfield,J.F. and Doudna,J.A. (2018) Programmed DNA destruction by miniature CRISPR-Cas14 enzymes. *Science*, **362**, 839–842.

2. Shmakov,S., Smargon,A., Scott,D., Cox,D., Pyzocha,N., Yan,W., Abudayyeh,O.O., Gootenberg,J.S., Makarova,K.S., Wolf,Y.I., *et al.* (2017) Diversity and evolution of class 2 CRISPR–Cas systems. *Nat. Rev. Microbiol.*, **15**, 169–182.

3. Zimmermann,L., Stephens,A., Nam,S.-Z., Rau,D., Kübler,J., Lozajic,M., Gabler,F., Söding,J., Lupas,A.N. and Alva,V. (2018) A Completely Reimplemented MPI Bioinformatics Toolkit with a New HHpred Server at its Core. *J. Mol. Biol.*, **430**, 2237–2243.

4. Song,Y., DiMaio,F., Wang,R.Y.-R., Kim,D., Miles,C., Brunette,T., Thompson,J. and Baker,D. (2013) High-Resolution Comparative Modeling with RosettaCM. *Structure*, **21**, 1735–1742.

5. Olechnovič,K. and Venclovas,Č. (2019) VoroMQA web server for assessing three-dimensional structures of proteins and protein complexes. *Nucleic Acids Res.*, **47**, W437–W442.

6. Lee,G.R., Won,J., Heo,L. and Seok,C. (2019) GalaxyRefine2: simultaneous refinement of inaccurate local regions and overall protein structure. *Nucleic Acids Res.*, **47**, W451–W455.
